# A critical step toward far-field laboratory diffraction contrast tomography in Laue focusing geometry

**DOI:** 10.1107/S1600576725001396

**Published:** 2025-03-13

**Authors:** Yubin Zhang, Adam Lindkvist

**Affiliations:** a Department of Mechanical Engineering Technical University of Denmark Kgs. Lyngby 2800 Denmark; b Department of Industrial and Materials Science Chalmers University of Technology SE 41296 Gothenburg Sweden; Ecole National Supérieure des Mines, Saint-Etienne, France

**Keywords:** far-field laboratory diffraction contrast tomography, dictionary-based branch and bound methods, grain indexing, strain analysis

## Abstract

Recent simulations have demonstrated the promising potential of far-field laboratory diffraction contrast tomography (FF-LabDCT) in the Laue focusing geometry for 3D mapping of grain-averaged deviatoric strain tensors, achieving a strain uncertainty as low as 1 × 10^−4^ [Lindkvist & Zhang (2022). *J. Appl. Cryst.***55**, 21–32]. The present work takes a critical step towards realizing this tool. It presents the first attempt at implementing FF-LabDCT using a Zeiss Xradia 520 Versa X-ray microscope, including both hardware and software development. A new algorithm, LabDBB, which adapts the previously developed dictionary-based branch and bound (DBB) principles, is implemented for indexing FF-LabDCT data. The performance of LabDBB is evaluated by comparing the indexed grain crystallographic orientations and center of mass positions with those obtained using near-field LabDCT within the same gauge volume of a fully recrystallized iron sample. Finally, the challenges related to fitting the grain-averaged deviatoric strain tensor using the current FF-LabDCT setup are identified. The strain analysis suggests that, once these challenges are addressed, a strain uncertainty as low as 2 × 10^−4^ could potentially be achieved. This work demonstrates the possibility for developing a tool capable of 3D grain-scale strain analysis with high strain precision in home laboratories.

## Abstract 2

A far-field laboratory diffraction contrast tomography (FF-LabDCT) technique is established and verified using conventional near-field LabDCT. Future directions to enhance FF-LabDCT as a versatile tool are outlined.

## 1. Introduction

Over the past decade, a new laboratory-based characterization technique, known as laboratory diffraction contrast tomography (LabDCT), has been developed for non-destructive 3D grain mapping of bulk samples (King *et al.*, 2013 ▸; McDonald *et al.*, 2015 ▸; Ganju *et al.*, 2023 ▸; Oddershede *et al.*, 2022 ▸; Fang *et al.*, 2023 ▸). This technique can be implemented in some conventional computed tomography systems and has now been commercialized by Xnovo Technology in collaboration with Carl Zeiss X-ray Microscopy. Using this commercial product, both the morphologies and the crystallographic orientations of grains in polycrystalline samples can be mapped in three dimensions. This technique is effective for recrystallized grains larger than 15–20 µm, offering a spatial resolution of ∼5 µm and an angular resolution of 0.1° (Sun *et al.*, 2022 ▸). LabDCT has already proven to be a valuable tool in material studies, including grain boundary wetting (Sun *et al.*, 2019 ▸), grain growth (Lu *et al.*, 2020 ▸), recrystallization nucleation (Lei *et al.*, 2021 ▸), corrosion (Zhao *et al.*, 2022 ▸) and plastic deformation (Kobayashi *et al.*, 2022 ▸; Nieto-Valeiras *et al.*, 2024 ▸).

**Figure 1:**
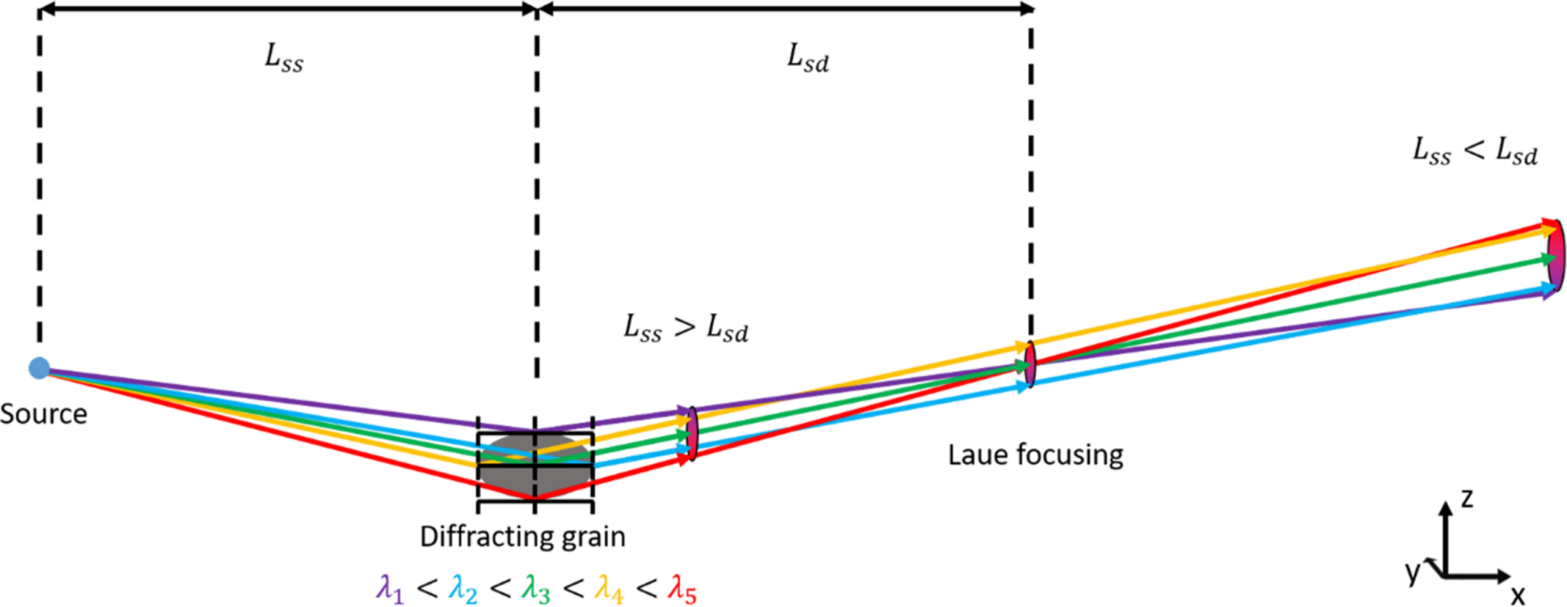
Schematic illustrating the principle behind LabDCT. The diffraction of various wavelengths from a single grain is depicted to demonstrate the Laue focusing effect. Spots are shown at three different positions to highlight the difference between two types of asymmetrical geometries and the Laue focusing geometry.

LabDCT utilizes a conical polychromatic X-ray beam generated from laboratory X-ray tubes, and its diffraction principle is based on the Laue focusing effect (Kvardakov *et al.*, 1997 ▸; Guinier & Tennevin, 1949 ▸; Stockmeier & Magerl, 2008 ▸) (see Fig. 1 ▸). In this process, divergent X-rays with different energies from a point source are diffracted by the parallel crystallographic lattice planes of a grain, producing a focused diffraction spot at a distance roughly equal to that between the sample and source. LabDCT adopts principles from its predecessors synchrotron DCT and 3D X-ray diffraction (3DXRD) [also known as high-energy X-ray diffraction (Poulsen, 2012 ▸; Li *et al.*, 2012 ▸; Reischig *et al.*, 2013 ▸)], such as utilizing tomographic data acquisition routines and diffraction spots for analyzing 3D grain structures. Therefore, it is natural to assume that LabDCT, similar to 3DXRD, may be operated in different modes: a near-field (NF) mode for resolving grain shapes with a high-resolution detector and a far-field (FF) mode for strain analysis (Oddershede *et al.*, 2010 ▸). Though commercial LabDCT supports only NF-LabDCT, the present authors have recently started exploring the possibility and potential of FF-LabDCT. Note that the terms near-field and far-field in this context have slightly different meanings than those used for synchrotron techniques. At synchrotrons, parallel beams are used, and these modes are defined by the sample-to-detector distance (*L*_sd_). In contrast, LabDCT employs a conical beam, with the modes determined by both the sample-to-detector and the sample-to-source (*L*_ss_) distances. Under Laue focusing conditions, these distances are equal. In the FF Laue focusing condition, the diffraction spots appear as distinctly sharp peaks and do not contain grain shape information, which makes them particularly good for grain-averaged strain analysis. Recent theoretical analysis has demonstrated that FF-LabDCT can be effectively used for grain-averaged strain analysis, achieving a potential strain uncertainty as low as 1 × 10^−4^, a marked improvement compared with 5 × 10^−4^ for NF-LabDCT (Lindkvist & Zhang, 2022 ▸).

On the basis of these simulated results, it appears that *L*_ss_ is the most critical parameter for strain resolution. This is primarily due to the assumption used in strain fitting, which posits that the center of mass of the diffraction spot corresponds to the diffraction vector determined at the grain’s center of mass. With a larger *L*_ss_, the beam achieves greater parallelism, thereby reducing the energy range of X-rays diffracting from each individual grain. This energy variation is illustrated in Fig. 1 ▸. Notably, all X-rays diffracting from the same plane (depicted by the black horizontal lines on the grain in Fig. 1 ▸) have different wavelengths, such as the yellow and blue X-rays, resulting in energy variation among the X-rays hitting different parts of the diffraction spot. A larger *L*_ss_ reduces the energy range of the X-rays diffracting from each individual grain, resulting in less intensity variation within individual diffraction spots. These variations are otherwise influenced by differences in flux and detector quantum efficiency across different X-ray energies. This reduction better satisfies the assumption for grain-average strain fitting. The Laue focusing condition is optimal for similar reasons.

Though the NF-LabDCT setup is readily accessible in commercial systems with a standard LabDCT configuration, the FF mode requires new development. As *L*_ss_ and *L*_sd_ increase, a larger detector is necessary to maintain angular coverage and capture enough diffracted X-rays, as demonstrated in the simulated case. Consequently, a new experimental setup must be established. Additionally, increasing *L*_ss_ reduces the beam intensity at the sample, which can lower the diffraction signal and necessitate impractically long exposure times. Furthermore, on a larger detector with a large pixel size, the spots may appear smaller, making them harder to distinguish from high-frequency noise.

This work is the first initiative to experimentally implement FF-LabDCT in Laue focusing geometry through both hardware and software development, enabling evaluation of its feasibility for strain analysis in typical engineering materials. The hardware development is carried out within a commercial X-ray contrast tomography system equipped with a LabDCT module. Currently, the commercial software *GrainMapper3D* (Bachmann *et al.*, 2019 ▸) for analyzing NF-LabDCT data does not support non-standard detectors. Open-source MATLAB code (Fang *et al.*, 2022 ▸) has also been developed to process NF-LabDCT data using similar principles to the commercial software. However, this approach is very computationally intensive; as the algorithm searches the entire orientation space voxel by voxel within the 3D volume, it requires high-performance computing hardware, such as a graphics-processing unit, to run efficiently. Due to the unique diffraction principle of LabDCT, other existing software for analyzing 3DXRD or DCT cannot be directly applied. Therefore, in this work a new, low-computational-cost algorithm is developed to index the FF-LabDCT dataset. This algorithm is a forward simulation-based method, inspired by the recently developed dictionary-based branch and bound (DBB) method for indexing superimposed synchrotron Laue micro-diffraction patterns (Seret *et al.*, 2022 ▸). This algorithm searches only the orientation space and not the sample space. It can therefore be implemented even on a standard laptop.

To facilitate this initial implementation of FF-LabDCT, a fully recrystallized iron sample is used. Synchrotron measurements confirmed that the grains in this sample are essentially strain free, with strain variations on the order of 10^−5^ (Zhang *et al.*, 2022 ▸). Strain values determined by FF-LabDCT that deviated from this order are considered measurement uncertainties. By analyzing the results, challenges related to practical strain fitting and the potentially achievable lowest strain uncertainty using FF-LabDCT are discussed. Suggestions for future improvements to make FF-LabDCT a useful tool for local strain/stress analysis with high strain precision are provided.

## 2. Experimental

### 2.1. Materials

The material used in this study was fully recrystallized pure iron (99.95 wt%) with a nearly random texture. The sample was cut into a needle shape with a diameter of approximately 0.6 mm in the examined region using electrical discharge machining, and then subjected to annealing at 850°C for 2 h and electropolishing. The average grain size of the sample was about 75 µm (Zhang *et al.*, 2022 ▸).

### 2.2. Experimental setup

**Figure 2:**
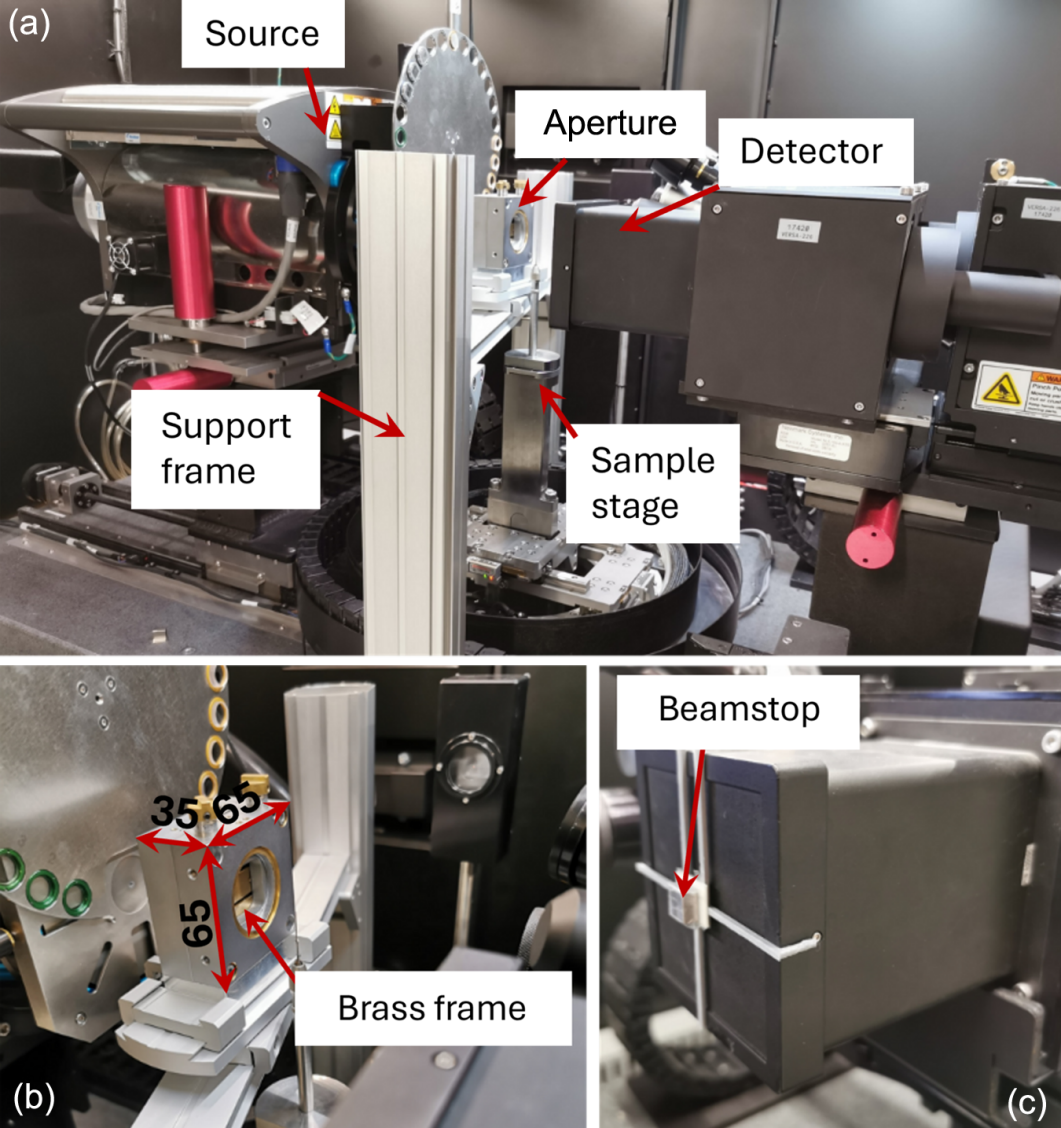
Photographs showing (*a*) the complete FF-LabDCT setup, (*b*) a close-up view of the aperture and (*c*) the detector along with the beamstop. The 2 mm-thick tungsten slits are behind a brass frame and are therefore not visible in (*b*).

The experiment was conducted using a Zeiss Xradia 520 Versa X-ray microscope equipped with a LabDCT module, which is designed for standard NF-LabDCT. For the present NF-LabDCT measurement, a 375 × 375 µm aperture and 2.5 × 2.5 mm beamstop in front of the 2048 × 2048 detector with an effective pixel size of 3.36 µm were used. For FF-LabDCT, a different detector with an alternative set of optics – increasing the effective pixel size by a factor of 10 – was used, which is available within the same microscope. However, since the FF setup is not standardized in this commercial system, specialized hardware was required. Specifically, a manually tunable external aperture was installed to limit the gauge volume [see Figs. 2 ▸(*a*) and 2 ▸(*b*)]. The aperture consists of two sets of 2 mm-thick tungsten slits with sharp edges and polished flat surfaces, attached to 5 mm-thick brass gliding frames in the center region. These slits were manually fine-tuned to adjust their positions and opening widths [see Fig. 2 ▸(*b*)] and were arranged horizontally and vertically to confine a box-shape beam. The aperture was aligned using the FF low-resolution detector as accurately as possible by eye (see Fig. S1 in the supporting information). The aperture was placed as close to the sample as possible to facilitate tuning. The precise position of the aperture relative to the sample was not measured, as the key parameter is the illuminated volume at the sample, which was determined from the detector image. Additionally, a custom-made lead beamstop, measuring 10 × 10 × 5 mm, was attached to the larger detector to prevent oversaturation [see Fig. 2 ▸(*c*)].

For the NF scan, both *L*_ss_ and *L*_sd_ were set to 13 mm. For the FF scan, these distances were increased to 100 mm. This distance was chosen to balance the X-ray intensity and strain resolution. Due to the constraints of the aperture support frame, a smaller *L*_ss_, like 50–75 mm, is not feasible in this setup. The exposure time was 200 s for the NF scan and 750 s for the FF scan. Both scans consisted of 121 projections, collected at 3° intervals as the sample was rotated through a full 360°, with the first and last projections taken at the same angle. A detector binning factor of 2 was applied for both scans to enhance the signal-to-noise ratio. In the FF case, this resulted in an effective pixel size of 67.2 µm, which is comparable to the average grain size of the sample. In line with the procedure for commercial LabDCT, several reference projections were also collected without the sample during the NF scan. However, this was not done for the FF scan, as a different background removal procedure was employed (see Section 2.3). The scans were performed sequentially without removing the sample, ensuring significant overlap of the scanned volumes in the central gauge region. However, the aperture was adjusted to examine a smaller sample portion in the FF case to limit the number of spots overlapping. This adjustment resulted in a smaller scanned volume with a height of ∼400 µm (see Fig. S1) for the FF scan, compared with ∼600 µm for the NF scan.

### 2.3. Data analysis

The NF data were processed using the commercial software *GrainMapper3D*, developed by Xnovo Tech. A/S, following their standard procedure. First, the diffraction images were normalized by dividing the value of each pixel by that of the corresponding pixel in the closest reference projection, and then a rolling median filter was applied across the 11 nearest projections. The diffraction spots were then segmented on the basis of the processed images using a line-segmentation algorithm as implemented in *GrainMapper3D*. The segmented projections from the NF data were reconstructed using the Fast Geometric Indexing algorithm (Bachmann *et al.*, 2019 ▸). The indexing of the NF data was performed using reflections from the first three {*hkl*} families, with completeness tolerances set to a minimum completeness of 40% and a trust completeness of 90%.

A different processing routine was applied for the FF data. First, the projections were normalized to account for variations in the source flux over time. A small 10 × 10 pixel region at the corner of the projections, which did not contain any spots, was selected to determine the normalization weights. This intensity variation was attributed to fluctuation in the source flux. The median of all the normalized projections was then used as a background and subtracted from each projection. A median filter was subsequently applied to the background-corrected data to remove high-frequency noise, enabling better segmentation of all the diffraction spots using a single intensity threshold.

The processed FF data were then indexed using a customized algorithm, LabDBB, which was modified from a previously developed DBB algorithm for micro-beam Laue diffraction. The LabDBB algorithm and its implementation are detailed in the following two subsections.

#### 2.3.1. LabDBB method

While the principles of the DBB method are detailed by Seret *et al.* (2022 ▸), a brief summary is provided here. The DBB method indexes grains by comparing theoretical diffraction vectors, calculated from a list of dictionary orientations, with those determined from experimental diffraction spots in single Laue diffraction images. A good candidate orientation from the dictionary, known as an orientation branch, is identified if a certain number of diffraction vectors from this orientation match some of the experimental vectors within an upper-bound deviation angle, which is determined by the dictionary branch resolution. The candidate orientations are then refined to determine whether they are true or false according to additional threshold criteria.

To accommodate the different data acquisition routine and diffraction principles of FF-LabDCT, two important modifications were made. First, the box beam used for FF-LabDCT illuminates multiple grains simultaneously, meaning that the diffraction center for each grain can no longer be assumed to originate from a single point as is the case in synchrotron micro-beam Laue diffraction (Seret *et al.*, 2022 ▸). Also, the grain position changes during sample rotation. Therefore, the grain’s center of mass was fitted along with its orientation based on the matched pairs of diffraction vectors. During this fitting process, the matched pairs of diffraction vectors were updated relative to the center of mass. Due to the grain’s center of mass shift from the origin, the upper-bound deviation angle in the original DBB method (determined by the branch resolution) (Seret *et al.*, 2022 ▸) was updated to account for the maximum angular deviation of the diffraction vector introduced by this shift. Secondly, the theoretical diffraction vectors were extended beyond a single diffraction image for synchrotron Laue diffraction to include all the diffraction images collected during sample rotation.

#### 2.3.2. Implementation

Similarly to the NF-LabDCT reconstruction, a completeness value is calculated to evaluate the quality of the indexing, determined by the ratio between the numbers of experimentally observed and theoretically expected diffraction spots. To expedite the indexing process, multiple indexing rounds were implemented, prioritizing large grains by setting a higher intensity threshold and a larger completeness value. The diffraction spots associated with these well indexed grains were then removed from the experimental spot list for the subsequent indexing round. For a fixed intensity threshold, the completeness threshold values gradually reduced to allow smaller grains to be indexed. After this, a new, lower intensity threshold was introduced to include weaker spots. This approach speeds up the process by reducing the number of candidate orientations that require further time-consuming refinement of the orientation and center of mass.

For this sample, the intensity threshold was set to be 200, 100, 60 and 40, below which a significant amount of background noise will be included. The completeness threshold for searching candidate orientations was set to range from 0.8 down to 0.4 in steps of 0.1.

For the indexing, the first three {*hkl*} families were used to compute the theoretical diffraction vectors, while the first ten {*hkl*} families were used to search diffraction spots associated with the indexed orientation. This is because incorporating spots from additional {*hkl*} families helps to improve the strain accuracy. The final indexed orientation required at least 150 experimental diffraction spots, with their diffraction vectors deviating by no more than 0.15° from the corresponding theoretically predicted ones, across all these ten {*hkl*} families. The lower spot number threshold was set to ensure the inclusion of small grains, which typically yield fewer spots, predominantly from low-order {*hkl*} families with high structure factors (Fang *et al.*, 2020 ▸). The deviation angle was determined by the maximum spot deviation from its expected position (4 pixels in this work, see Section 3.3). A slightly large angle of 0.5° was used for initial indexing prior to fitting the detector geometry. The detector geometry and the source position were then fitted following the procedures described by Fang *et al.* (2022 ▸) using the initial indexing of the large grains. A branch resolution of 2.5° was applied to generate the dictionary orientations using *MTEX*, resulting in 39 565 orientations within the fundamental zone.

The LabDBB algorithm was implemented in MATLAB (https://github.com/YubinZhang/Indexing_ff_LabDCT_DBB), and the code is compatible with parallelization computing. With a Zbook Fury 16G10 workstation laptop, indexing took about 63 min.

## 3. Results and discussion

### 3.1. Validation of the FF-LabDCT results

**Figure 3:**
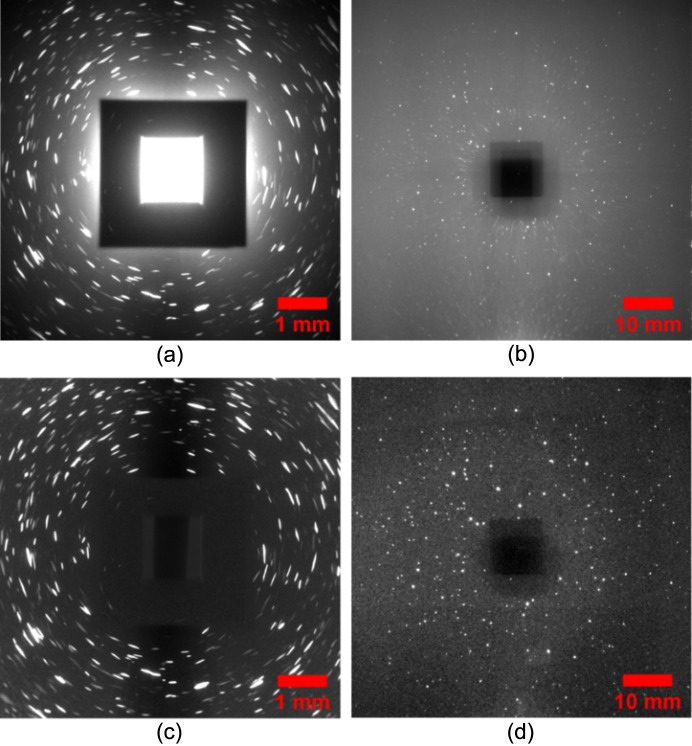
Typical projections from the (*a*), (*c*) NF and (*b*), (*d*) FF scans. The projections in (*a*) and (*b*) show unprocessed data using the same gray­scale, while (*c*) and (*d*) display processed data using background division and subtraction, respectively, with different grayscales applied.

Typical raw and background-removed diffraction images for both cases are shown in Fig. 3 ▸. The diffraction spots in the FF-LabDCT images appear rounder than those in the NF-LabDCT images, primarily due to the large detector pixel size used. A much lower background level is observed around the beamstop region in the FF case, which enables a more efficient subtraction routine for background correction, in contrast to the division routine used for the NF case. The subtraction routine preserves the true spot intensity and is more effective for retaining weak diffraction spots (Lindkvist *et al.*, 2021 ▸), making it more suitable for processing the FF data. A slightly elevated background level is observed on the left side of the image in Fig. 3 ▸(*b*), likely due to a small misalignment of the sample and aperture in the horizontal direction (see Fig. S1). Since this is effectively removed as a constant background, it is not critical for spot segmentation.

**Figure 4:**
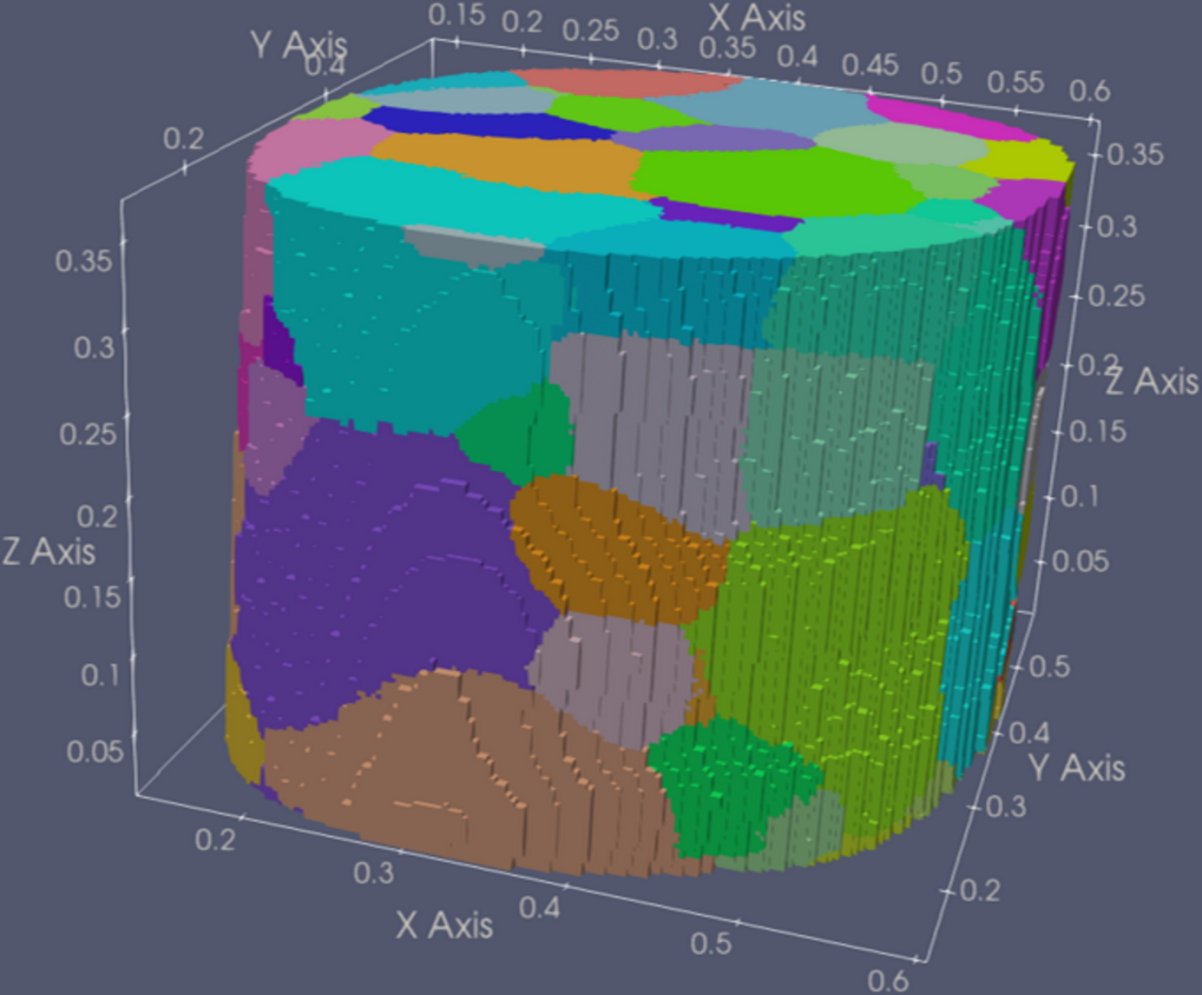
3D reconstruction of the NF dataset, with colors representing the orientation along the sample rotation (*Z*) axis.

In total, 172 grains were found from the NF data. A detailed comparison of the grain positions between the two indexed results revealed that the gauge volume of the FF scan deviated from that of the NF scan, with the common overlapping region located at the bottom of the NF volume. Since the two setups were aligned independently, this deviation is probably due to poor calibration of the system at large *L*_ss_ and *L*_sd_. However, this is not critical, as the data processing was conducted separately, and any relative displacement of the setup origins was accounted for and eliminated during the comparison. Within the common region, ∼370 µm in height, 96 grains are present in the volume reconstructed from the NF data (see Fig. 4 ▸). These 96 grains will be used for comparison with the FF-LabDCT results.

**Figure 5:**
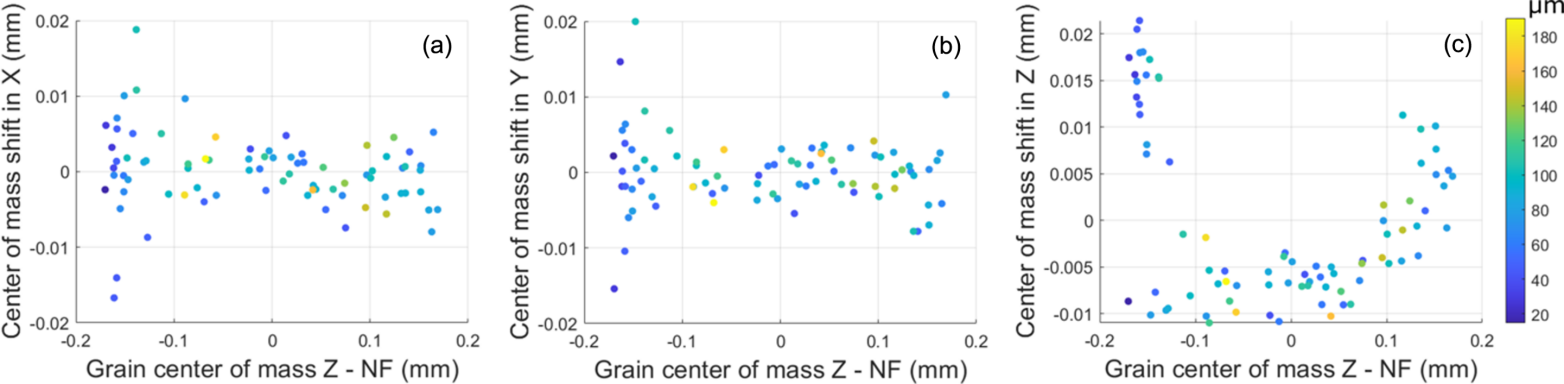
Comparison of grain positions between the NF and FF results for the 80 common grains. (*a*)–(*c*) Center of mass shifts in the *X*, *Y* and *Z* directions, respectively, between the two results as a function of the grain’s *Z* coordinate in the NF results. The data are colored according to grain size.

A total of 86 grains were indexed using LabDBB for the FF case. It is found that 80 out of these 86 indexed orientations can be matched to one of the 96 grains from NF-LabDCT, with a misorientation of less than 0.1°. The center of mass difference between these matched pairs is about 5–10 µm for grains in the central gauge volume, but it is larger near the gauge surface, especially along the *Z* direction (see Fig. 5 ▸). There is no clear trend between the center of mass shift and the grain size. The comparatively large uncertainty along the *Z* direction is probably due to the mismatch between the two gauge volumes. Additionally, for grains partly illuminated by the conical incoming beam near the gauge surface, the illuminated volume changes during sample rotation, affecting the resulting grain center of mass.

**Figure 6:**
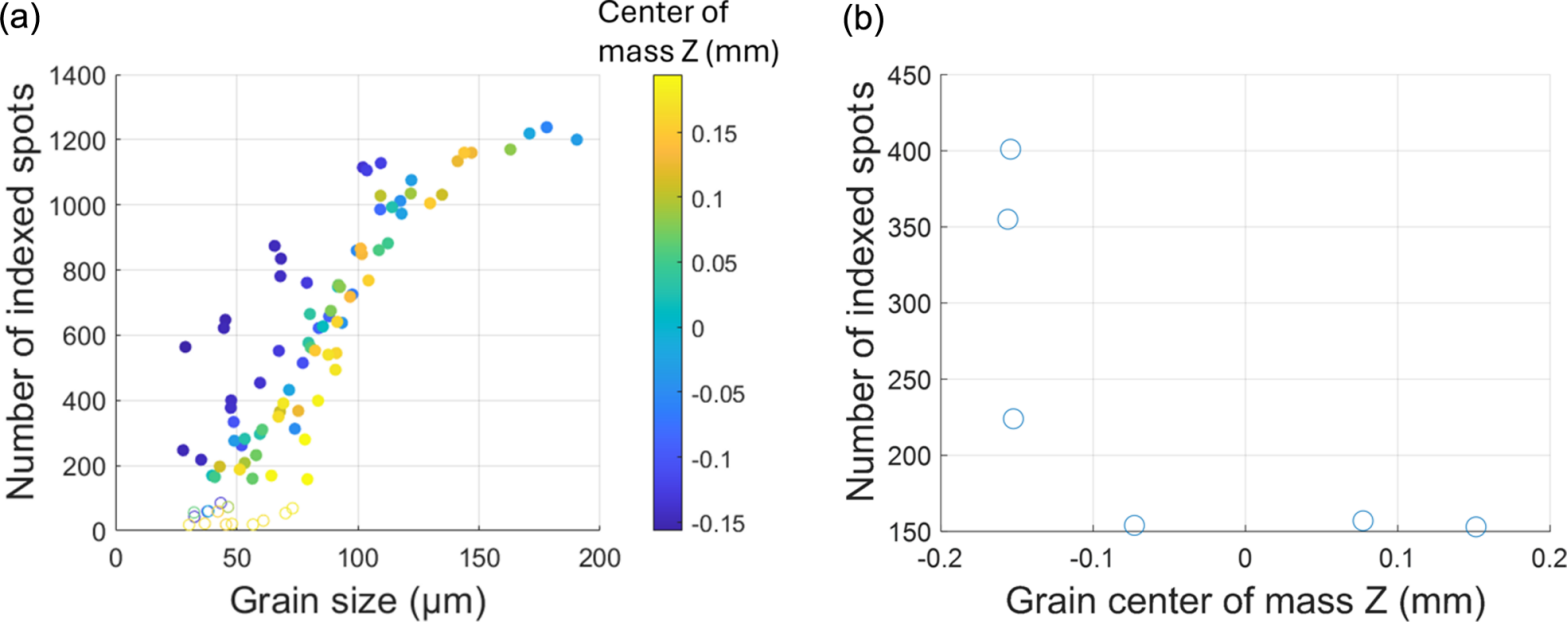
(*a*) Number of indexed spots as a function of grain size. Open points represent missing grains, while solid points represent indexed grains. The points are colored according to the grain center of mass *Z* coordinate in the NF results. (*b*) Number of indexed spots for the six absent grains within the NF-LabDCT volume, plotted as a function of the center of mass *Z* coordinate in the FF-LabDCT volume.

The number of indexed spots for each grain as a function of grain size, determined from the NF data, is shown in Fig. 6 ▸(*a*). For the large grains, more than 1200 spots are found from the first ten {*hkl*} families, while only ∼150 are found for smaller grains. A clear trend of decreasing diffraction spot count with decreasing grain size is evident. Grains deviating from this general trend are mainly from the mismatched bottom gauge volume [shown as blue points in Fig. 6 ▸(*a*)]. The actual grain size illuminated in the FF case is likely to be bigger than it appears in the NF case, where the grains are only partially illuminated. Excluding these blue points, the general trend suggests a critical grain size of ∼50 µm for the FF case, above which grains can be reliably mapped by FF-LabDCT.

Additionally, on the basis of the grains indexed from the NF data, the diffraction spots associated with missing grains were tracked. Fewer than 100 spots are found for these non-indexed grains [shown as open circles in Fig. 6 ▸(*a*)]. These grains are either comparatively small or located close to the upper gauge volume [shown as yellowish open circles in Fig. 6 ▸(*a*)], which may have been only partly illuminated in the FF case.

For the six grains indexed by FF-LabDCT but not in the NF-LabDCT volume, there are more than 150 spots associated with these grains, even after excluding spots that overlap with other grains [see Fig. 6 ▸(*b*)]. Notably, the grains near the bottom of the gauge volume are associated with more than 200 unique diffraction spots. The absence of these three grains within the NF-LabDCT volume is probably due to the volume mismatch. The other three grains, associated with ∼150 spots, may be false positives.

Given that NF-LabDCT does not necessarily provide the actual ground truth, this comparison suggests that the indexing results from the current FF-LabDCT data are satisfactory overall. Additionally, the reduced computing resources required for LabDBB suggests that its indexing principles may be adapted for NF-LabDCT to speed up the process in the future.

### 3.2. Efficiency of FF-LabDCT

It is observed that only grains larger than 50 µm can be reliably mapped with this first FF-LabDCT setup in Laue focusing geometry, even with a long exposure time of 750 s. The intensity is typically inversely proportional to the square of the distance from the source (*i.e.*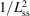). An increase in distance from 13 to 100 leads to an intensity drop by a factor of ∼60. Nonetheless, a detector with a pixel size 10 times larger results in a 100-fold increase in the area for accepting photons per pixel, which somewhat compensates for the intensity drop due to the increased *L*_ss_. Even so, given the critical grain size of ∼20 µm for the NF case, the critical size of 50 µm in the FF case implies that the NF setup is 15 times (2.5^3^) more efficient than the FF setup. When combined with 3.75 times longer exposure time, this results in the FF setup being ∼1/55 as efficient as the NF.

The drop in efficiency is partly attributed to the longer interaction path between the photons and air in the FF case. For X-rays with energy in the range 15–60 keV, which is the primary range for LabDCT (Lindkvist *et al.*, 2021 ▸), 200 mm of air can lead to an absorption of 27 to 4%. Additionally, the quantum efficiency of the scintillators in the large detector may be comparatively poor. This analysis suggests that, for grains of 20 µm, a 50 times longer exposure time would be required, which is clearly impractical with the current commercial setup. A state-of-the-art photon counting detector can be used to remedy this issue, as discussed further in Section 3.4.

### 3.3. Strain analysis

In this section, several factors that may affect strain fitting are discussed prior to presenting the strain fitting results. Considering the large effective detector pixel size used for the FF scan (67.2 µm), the fitting accuracy of 5–10 µm is likely to be acceptable for the present FF-LabDCT. Improved calibration between the NF and FF setup could be beneficial for incorporating the center of mass determined from the NF data to better evaluate strains and verify any differences. The above analysis also reveals that grains that are partly illuminated may lead to reduced strain fitting accuracy, as the center of mass of the illuminated regions changes during rotation.

**Figure 7:**
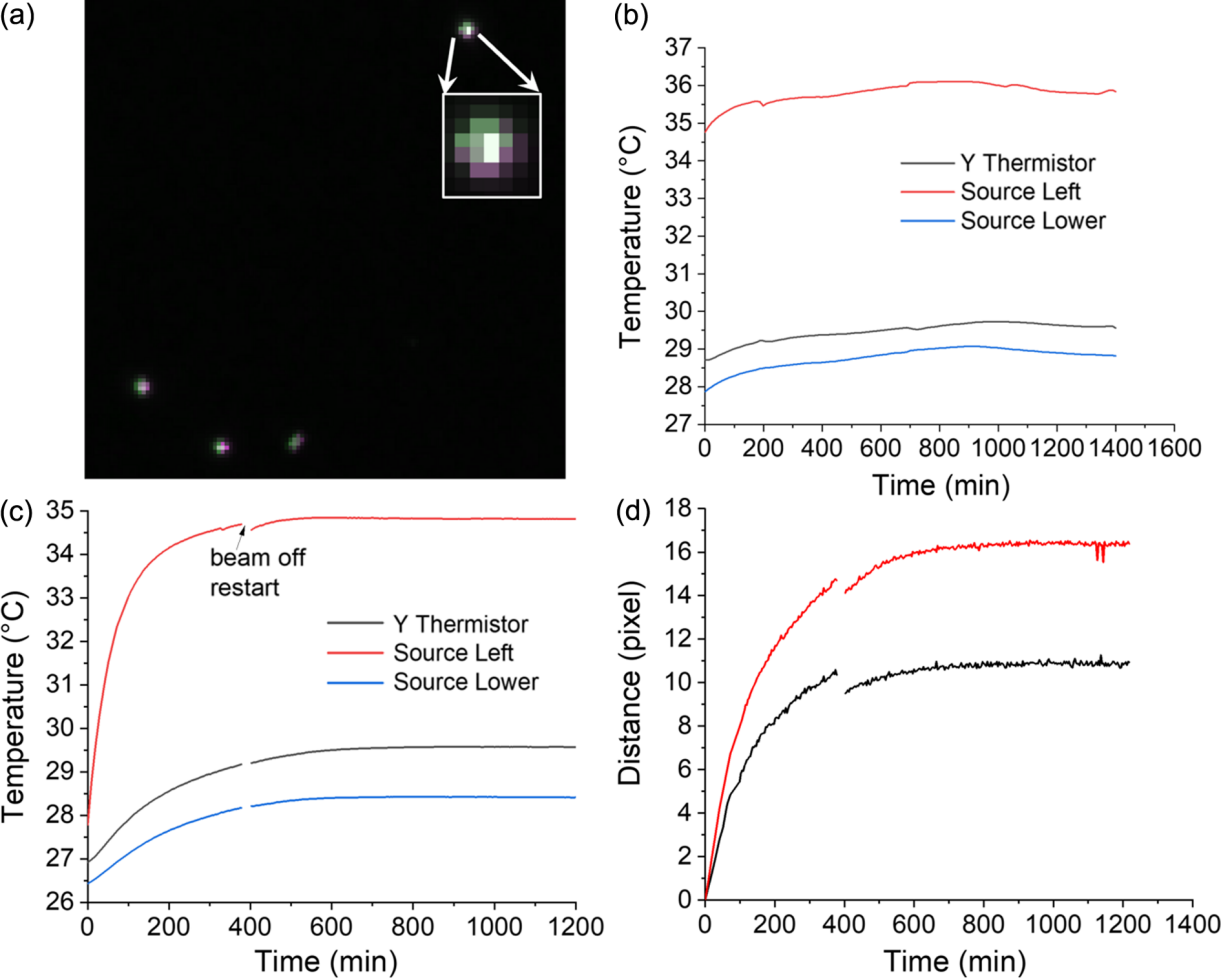
Impact of system thermal stability. (*a*) Magnified view of a composite RGB image showing the first and last diffraction images of the FF-LabDCT dataset, collected at nominally the same sample position, overlaid in different color bands. Green, magenta and gray colors represent the pixels present only in the first image, only in the last image and commonly in both images, respectively. The inset provides an even more enlarged view of the top spot. (*b*) Temperature profile associated with each diffraction image in the FF-LabDCT dataset. (*c*) Temperature profile and (*d*) spot shift distance (in pixels) for two example diffraction spots for a dataset collected at the same sample position over a long acquisition period.

Since strain fitting relies on the position of the diffraction spots, their stability against thermal and mechanical drift of the system during a long scan period is a concern. The first and last diffraction images were nominally from the same sample position after a full 360° rotation. A slight shift of ∼1 pixel (67.2 µm) was observed in all the diffraction spots between the two projections, as shown in the magnified view in Fig. 7 ▸(*a*). This shift is likely to be due to system temperature variations, which were recorded by the commercial system during data collection [see Fig. 7 ▸(*b*)].

To further quantify this drift, a new series of diffraction data was collected while keeping the sample position unchanged, using only the NF detector without any pixel binning. The center of mass of two selected diffraction spots was then tracked as a function of collection time [see Fig. 7 ▸(*d*)]. A significant spot shift is observed at the beginning, before the system has thermally stabilized. A temperature change of 2°C, as measured by the system’s Y thermistor at the very beginning, can induce a 10–15 pixel shift (30–50 µm) in the diffraction spots. The system stabilizes about 500 min after the instrument doors are closed, after which only subpixel shifts are observed. The higher temperatures recorded by the Y thermistor are likely from a location close to the source, and the variation among the three temperature measurements suggests a temperature gradient from the source to the surrounding environment. Evidently, restarting the source affects its temperature. In addition, the better correlation between the Y thermistor readout and spot displacement during beam restarts indicates that the source temperature is the most critical factor. The correlation between temperature changes close to the source and spot position shifts indicates a shift of ∼4 pixels per °C. Applying this correlation to the FF data suggests a 0.3 pixel shift within the maximum 1.5°C variation during the scan, which is smaller than the shifts shown in Fig. 7 ▸(*a*). Some additional drift (*e.g.* due to sample rotation) may have occurred.

**Figure 8:**
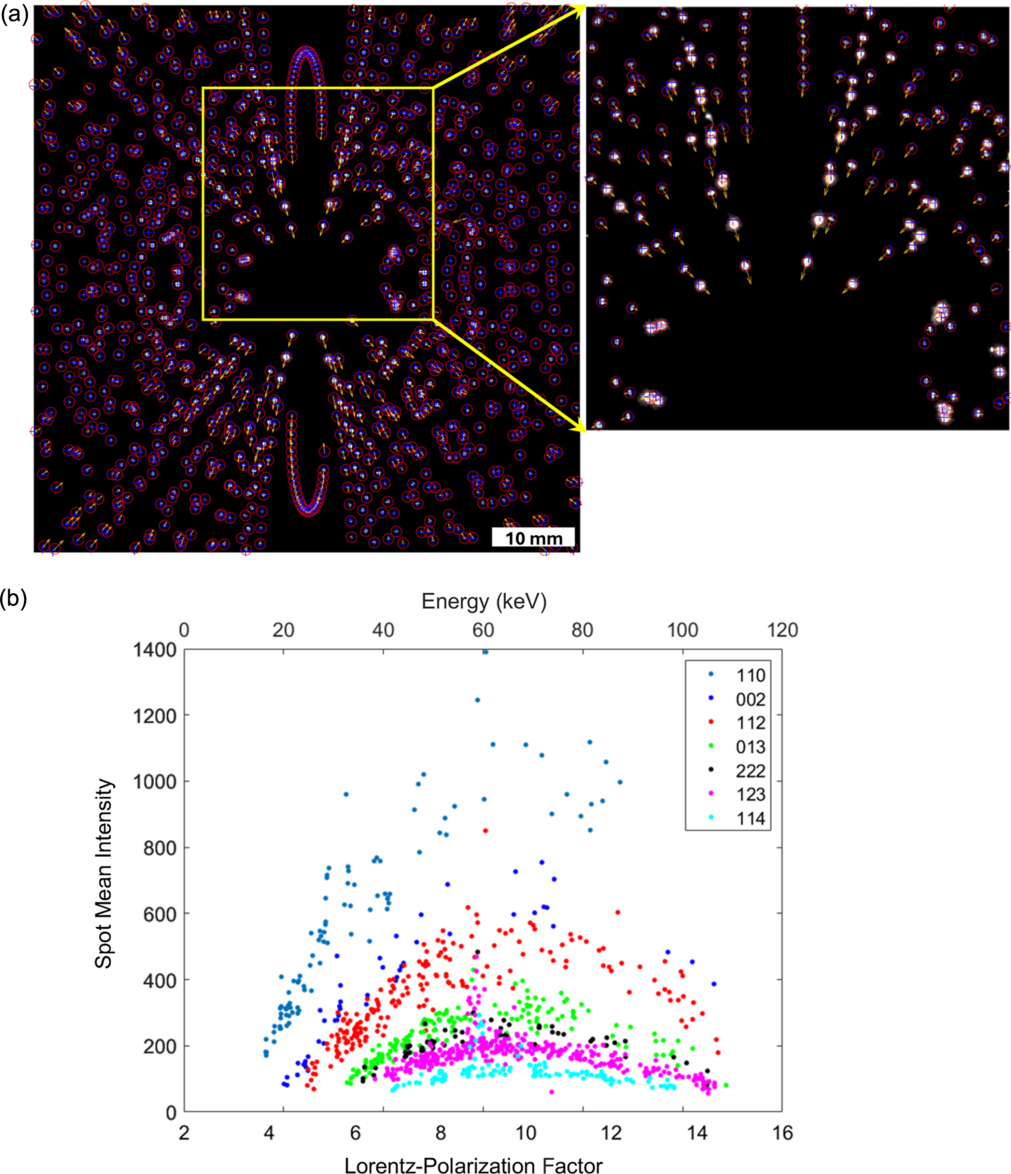
(*a*) Comparison between experimentally observed diffraction spots (shown as white pixels and red circles) and their theoretical positions (marked by blue crosses) for a large grain. This image consolidates all the diffraction spots from the first 10 {*hkl*} families observed in the 121 diffraction images. The orange arrows indicate the direction and relative magnitude of the displacement between each pair of spots. (*b*) Correlation between spot mean intensity and Lorentz–polarization factor/X-ray energy for the spots shown in (*a*). Note that {220}, {330} and {004} are excluded due to overlap with their parallel lower-index *hkl* reflections.

To further assess the quality of the FF-LabDCT data for strain analysis, all diffraction spots originating from a large grain were collected and included in a single projection image [see Fig. 8 ▸(*a*)]. The expected positions of the diffraction spots, based on the indexed grain’s center of mass and orientation, were overlaid on the image. Systematic shifts between the experimentally observed and theoretical strain-free spot positions, ranging up to 3–4 pixels, are observed in the central and four corner regions. These spot shifts could not be corrected by adjusting the detector position and are probably due to imperfect detector distortion correction in the commercial system or other unknown factors.

Furthermore, significant variation in spot intensity across different {*hkl*} families is observed for this grain, mainly due to differences in structure factors [see the magnified view in Fig. 8 ▸(*a*)]. The diffraction spots generally appear stronger in the central regions of the detector, primarily because of the higher Lorentz–polarization factor, [1 + cos^2^(2θ)]/sin(2θ), at lower 2θ angles [see Fig. 8 ▸(*b*)]. While the spots are well separated among *hkl* reflections, the deviation from a linear intensity relationship at higher Lorentz–polarization factors is attributed to the lower X-ray flux of the source tube and the comparatively lower quantum efficiency of the detector at higher X-ray energies (Fang *et al.*, 2020 ▸). The comparatively higher X-ray energies associated with these spots at lower 2θ angles also lead to deeper penetration of the diffracted X-rays into the scintillator and thus larger impacted regions. The difference in penetration depth between the detector central and outer regions may introduce additional variations in the spot center of mass. For example, at a 2θ angle of 10°, a penetration depth difference of 100 µm can result in an ∼18 µm shift in the spot center of mass, and a deeper penetration into the scintillator can make the spot appear farther from the center than expected. Additionally, the angular resolution in the central region is comparatively worse, potentially leading to more spot overlap. All these factors may influence the determination of the diffraction vectors.

Despite these challenges, strain fitting analysis was carried out using the present FF-LabDCT data. The fitted strain tensor for the grain shown in Fig. 8 ▸(*a*) is [−2.4 0.1 −0.4; 0.1 −2.4 −0.1; −0.4 −0.1 4.8] × 10^−4^. Since the sample is strain free, the non-zero normal strain components (>1 × 10^−4^) determined here are attributed to errors, primarily stemming from detector distortion. The comparatively large tensile strain along the *Z* direction primarily results from the pronounced downward deviations of the observed spot positions compared with the theoretically predicted ones for a strain-free case near the vertical center, caused by detector distortion. These spots are mainly from diffraction vectors close to the rotation axis *Z*. In contrast, the spots corresponding to diffraction vectors parallel to the *X* and *Y* directions are near the horizontal center, where good alignment between the observed and theoretical predictions is observed. The compressive strains along the *X* and *Y* directions are therefore primarily a consequence of volume constraints in the deviatoric strain tensor fitting.

This pattern is consistent across all the grains, as shown in the box chart in Fig. S2, illustrating the distribution of each strain component for 80 commonly observed grains. This consistency further supports the fact that the non-zero normal strain components are primarily dominated by detector distortion. Excluding a few outliers, the full width at half-maximum of each strain component is about 2 × 10^−4^, aligning with the strain uncertainty level determined from simulated data. Combined with the nearly zero shear strains, as expected for the present sample, these results suggest that a similarly low level of strain uncertainty could be achievable once the detector distortion issues are resolved. Notably, this strain uncertainty level is lower than the estimated value based on a single-pixel position deviation of the spots, calculated as 1 pixel × 67.2 µm pixel^−1^/100 mm = 6.7 × 10^−4^. This is because multiple diffraction spots from the same (*hkl*) plane (up to 20) and numerous unique (*hkl*) planes (ranging from 15 to 150) are included in the strain fitting process. Different spot shifts for an (*hkl*) plane, observed at different rotation angles and thus associated with different X-ray energies, are constrained to minimize the spot shift for this (*hkl*) plane. A similar constraint is applied across different (*hkl*) planes. As a result, spot shifts caused by thermal instability and different penetration depths may be averaged out when these shifts occur in random directions. If detector distortion issues are addressed, analysis of spot shifts in relation to different energy levels and different (*hkl*) planes could provide valuable insights into their impact on the strain fitting results.

A similar analysis was conducted for the NF data, only for grains with an average completeness larger than 95% and size larger than 25 µm. An average strain close to zero is obtained for all strain components (see Fig. S3), albeit with a larger strain uncertainty of 5–6 × 10^−4^. This uncertainty aligns reasonably well with values determined from simulated data under the same setup geometry (Lindkvist & Zhang, 2022 ▸). However, these results still require proper verification in the future. Despite this, the analysis demonstrates the potential advantage of strain analysis using FF-LabDCT over NF-LabDCT.

### 3.4. Future directions

LabDCT is currently the only method available to reveal the 3D grain structure (including morphology, position and orientation) of fully recrystallized crystalline materials in home laboratories using micro-focus X-ray sources with a tungsten target. This capability is enabled by the use of a continuous polychromatic X-ray spectrum from laboratory sources that compensates for the low intensity of monochromatic X-rays from characteristic lines, which is 10–15 orders of magnitude lower than that of synchrotron sources (Feser *et al.*, 2015 ▸). Even though the X-ray intensity of the monochromatic characteristic X-rays of tungsten can be increased by boosting the input electron power by a factor of 10–100, resolving individual grains after collimation remains a significant challenge (Zhang *et al.*, 2014 ▸). An added advantage of using a polychromatic beam over a monochromatic one is the reduction in the required number of projections by a factor of 10–30, enabling significantly longer exposure times per projection — critical for resolving smaller grains. Moreover, the use of high-energy X-rays, up to 150 keV, facilitates the study of high-*Z* materials (Lindkvist *et al.*, 2021 ▸). Thus, implementing FF-LabDCT for grain-scale strain analysis with high strain accuracy using polychromatic X-rays, integrated with NF-LabDCT within the same instrument for comprehensive 3D analysis, is a promising way forward. Its potential for commercialization could allow broader accessibility worldwide, overcoming the limited availability at a few synchrotron facilities.

Nonetheless, all the artifacts discussed in Section 3.3 must be eliminated in future strain fitting. While the thermal stability issue can be relatively easily addressed with longer waiting times and more stable sample mounting – such as a stronger magnetic kinematic stage or mechanical fixture – and the energy-induced spot shift can be corrected through theoretical analysis, the most critical challenge with the present setup is detector distortion. A promising solution to this issue is the use of flat-panel detectors, such as those in certain Zeiss Versa or CrystalCT microscopes, which avoid optical components.

To make FF-LabDCT a more versatile tool, it is essential to extend the detection limit to smaller grain sizes. In this regard, exploring the use of liquid metal jet (David *et al.*, 2004 ▸) or linear accumulation X-ray (Yun *et al.*, 2017 ▸) sources, which provide 10–100 times higher intensity than the current tungsten-target micro-focus source, would be worthwhile. Recently, a laboratory FF-3DXRD setup using a liquid metal jet source has been established. It has been demonstrated that this setup is capable of indexing grains larger than 50–60 µm in Mg samples using a collimated monochromatic beam of In characteristic X-rays at ∼24 keV, with a typical *L*_ss_ of ∼800 mm and a strain resolution of ∼1 × 10^−4^ (Oh *et al.* 2024 ▸). Implementing FF-LabDCT with such a source at *L*_ss_ ≃ 100 mm would result in an intensity gain of 8^2^ = 64. Even for the monochromatic characteristic X-rays alone, this enhancement is expected to extend the detection limit to a significantly smaller grain size (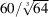 = 15 µm). Additionally, photon-counting detectors can improve the signal-to-noise ratio and reduce exposure time, while also eliminating the need for optical components, thus avoiding spatial distortion. Preliminary tests indicate that an Advacam ADVAPIX TPX3 detector achieves an exposure time reduction factor of ∼200 compared with the flat-panel detector in another Zeiss 520 Versa microscope.

Furthermore, with polychromatic X-rays, the exact energies for individual diffraction peaks are unknown, allowing extraction of only the deviatoric strain tensor, *i.e.* the lattice distortion (Lindkvist & Zhang, 2022 ▸). While this alone would mark a significant advancement, FF-LabDCT could be further enhanced to include the hydro­static component of the strain tensor using characteristic X-rays to resolve the lattice constant (Zhang *et al.*, 2014 ▸).

Aside from hardware improvements, further optimization of FF-LabDCT experiments is needed. Simulations indicate that increasing *L*_ss_ improves strain resolution (Lindkvist & Zhang, 2022 ▸). However, increasing *L*_ss_ would reduce the number of detected spots, which would be detrimental to strain accuracy. The effects of data acquisition parameters, including *L*_ss_, *L*_sd_, exposure time, detector pixel size and number of projections, should be investigated further to optimize strain fitting experiments. To this end, a more flexible motorized aperture setup should be designed to enable automated alignment of the aperture position and slit opening width, potentially by inserting apertures from the sides or top. This flexibility is necessary because, as the source moves farther from the sample, significantly smaller apertures are required if attached to the source to maintain a reduced illuminated volume at the sample. For instance, with the aperture located ∼5 mm from the source (as in the current NF-LabDCT commercial system) and at *L*_ss_ = 200 mm, a 7.5 µm slit on the aperture would be needed to confine a 300 µm illuminating volume height at the sample, which presents considerable manufacturing challenges.

In addition to the strain-free samples studied here, recrystallizing grains in partially recrystallized samples, where significant residual strains have been observed with a macroscopic correlation between strain tensor and sample directions (Lindkvist *et al.*, 2023 ▸), could be promising candidates for FF-LabDCT experiments. Measurements on samples under external load could be carried out to further validate the method, similar to that done recently with neutron diffraction tomography (Larsen *et al.*, 2024 ▸). *In situ* deformation rigs are already available for laboratory X-ray systems (Kobayashi *et al.*, 2022 ▸). In this context, the principles of the previously developed Laue-DIC digital image correlation method (Petit *et al.*, 2015 ▸) could be adapted to enhance strain resolution. Finally, a direct comparison with synchrotron measurements would be necessary to validate the technique.

With these improvements in place – particularly an efficiency gain of 1000× through the combined use of a commercially available more intense source and a photon counting detector – FF-LabDCT is expected to be able to measure grains as small as 10–15 µm, even at *L*_ss_ = 200 mm, with a strain resolution of 1 × 10^−4^. Combined with NF-LabDCT, this would make it a powerful tool for materials research in home laboratories.

## 4. Conclusions

FF-LabDCT was implemented with both hardware and software developments. The first FF-LabDCT dataset was collected from a pure iron sample with an average grain size of 75 µm and analyzed using a new algorithm, LabDBB. The indexing results were compared with NF-LabDCT results collected from roughly the same gauge volume. The comparison showed that, with the present FF-LabDCT setup, grains larger than 50 µm can be mapped reliably. Further examination of the indexed results revealed artifacts caused by system thermal instability and detector imperfections, which must be addressed to improve the accuracy of strain fitting with FF-LabDCT. Additionally, the impact of different X-ray energies on the position of the diffraction spots should also be considered. Once these challenges are addressed, a strain uncertainty as low as 1–2 × 10^−4^ could potentially be achieved with FF-LabDCT, in contrast to 5–6 × 10^−4^ for NF-LabDCT. On the basis of the valuable lessons learned, future directions for developing FF-LabDCT into a more versatile tool, in combination with NF-LabDCT, have been outlined.

## Supplementary Material

Suporting figures. DOI: 10.1107/S1600576725001396/nb5392sup1.pdf
