# Supplementary material for: A critical step toward far-field laboratory diffraction contrast tomography in Laue focusing geometry
Source: J Appl Crystallogr. 2025 Mar 13;58(Pt 2):447–57. doi: 10.1107/S1600576725001396 (PMC11957408; doi:10.1107/S1600576725001396)
Supplement: Supplementary file 1 [file j-58-00447-sup1.pdf]

JOURNAL OF APPLIED CRYSTALLOGRAPHY

Volume 58 (2025)

Supporting information for article:

A critical step toward far-field laboratory diffraction contrast tomography in Laue focusing geometry

Yubin Zhang and Adam Lindkvist

A critical step toward far-field laboratory diffraction contrast tomography in Laue focusing geometry

Yubin Zhang [a*], Adam Lindkvist [a,b]

[a] Department of Civil and Mechanical Engineering, Technical University of Denmark, Kgs. Lyngby, 2800, Denmark

[b] Department of Industrial and Materials Science, Chalmers University of Technology, SE 41296, Gothenburg, Sweden

Correspondence email: yubz@dtu.dk

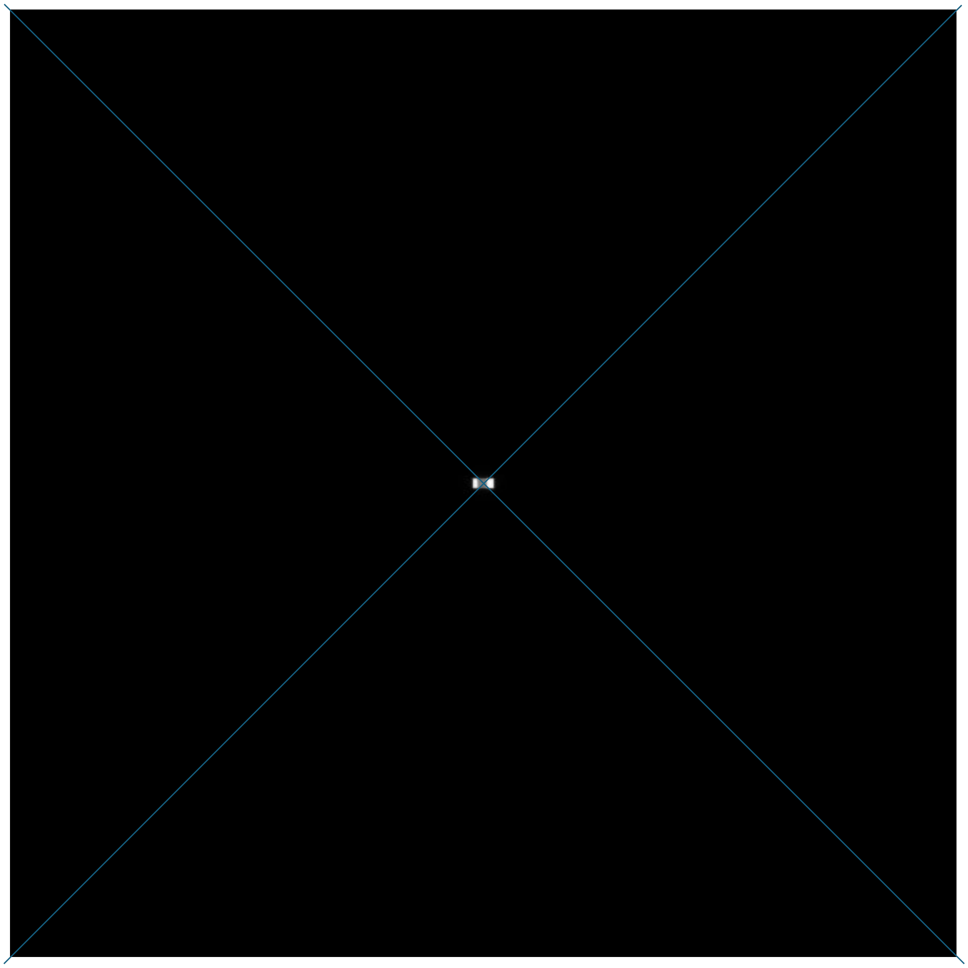

Figure S1. Detector image showing the direct beam passing through the aperture for the far-field setup, collected at L_{sd} of 110 mm.

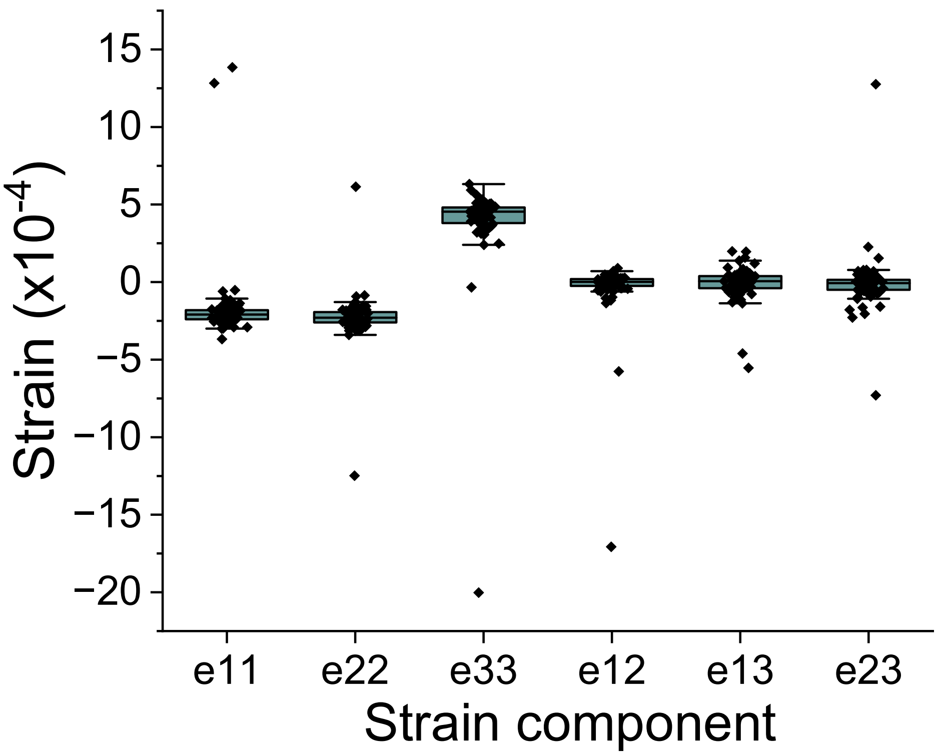

Figure S2. Box chart showing the distribution of each strain component from the fitted tensors for the 80 commonly observed grains.

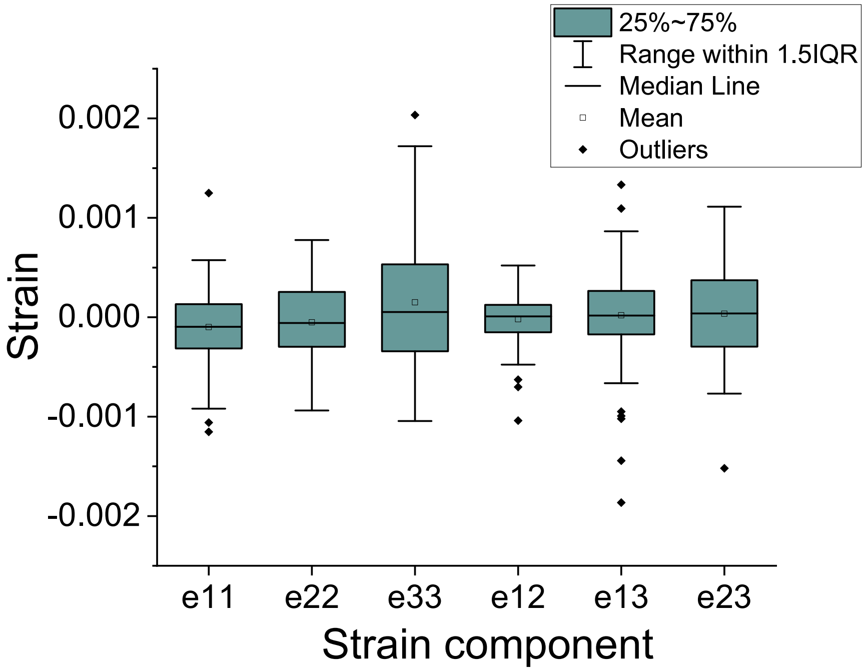

Figure S3. Distribution of strains for different components determined based on NF-LabDCT data. Only the grains with an average completeness larger than 95% and size larger than 25 μm are used.
